# Supplementary material for: Patient-Derived Colorectal Cancer Organoids Upregulate Revival Stem Cell Marker Genes Following Chemotherapeutic Treatment
Source: J Clin Med. 2020 Jan 2;9(1):128. doi: 10.3390/jcm9010128 (PMC7019342; doi:10.3390/jcm9010128)
Supplement: Supplementary file 1 [file jcm-09-00128-s001.pdf]

Table S1. Patient clinical information.

Patient	Overall Stage	Age	Gender	dMMR Status	Tumour Site	Tumour Type	Differentiation	Years from Surgery	Current Status	Time to Progression
58T	1	75	M	normal	sigmoid colon	adeno	poor	1.9	Alive, no recurrence	n/a
63T	1	79	F	normal	ascending colon	adeno	mod	1.0	Alive, no recurrence	n/a
30T	2	86	F	normal	ascending colon	adeno mucinous	mod	2.0	Deceased, second primary cancer	1.8
61T	2	87	F	normal	transverse colon	adeno	mod	2.0	Alive, no recurrence	n/a
38T	3	84	F	normal	sigmoid colon	adeno	mod	1.7	Alive, no recurrence	n/a
53T	3	81	M	normal	ascending colon	adeno	poor	1.1	Alive, no recurrence	n/a
54T	3	66	F	normal	caecum	adeno	poor	1.3	Alive, no recurrence	n/a
46T	3	74	F	normal	hepatic flexure	adeno	mod	2.0	Alive, no recurrence	n/a
78T	3	55	F	normal	rectum upper third (> 12cm)	adeno	mod	1.5	Alive, no recurrence	n/a
64T	4	41	F	normal	caecum	adeno	poor	1.8	Alive, with disease*	1.48
67T	4	38	M	normal	rectum upper third (> 12cm)	adeno	mod	1.8	Alive, no recurrence#	1.75
